# How Does it STAC Up? Revisiting the Scaffolding Theory of Aging and Cognition

**DOI:** 10.1007/s11065-014-9270-9

**Published:** 2014-08-21

**Authors:** Patricia A. Reuter-Lorenz, Denise C. Park

**Affiliations:** 1 Department of Psychology, The University of Michigan, 530 Church Street, Ann Arbor, MI 48109 USA; 2 Center for Vital Longevity, School of Brain and Behavioral Sciences, The University of Texas at Dallas, Richardson, TX 75235 USA

**Keywords:** Cognitive aging, Brain imaging, Scaffolding, Compensation

## Abstract

“The Scaffolding Theory of Aging and Cognition (STAC)”, proposed in 2009, is a conceptual model of cognitive aging that integrated evidence from structural and functional neuroimaging to explain how the combined effects of adverse and compensatory neural processes produce varying levels of cognitive function. The model made clear and testable predictions about how different brain variables, both structural and functional, were related to cognitive function, focusing on the core construct of compensatory scaffolding. The present paper provides a revised model that integrates new evidence about the aging brain that has emerged since STAC was published 5 years ago. Unlike the original STAC model, STAC-r incorporates life-course factors that serve to enhance or deplete neural resources, thereby influencing the developmental course of brain structure and function, as well as cognition, over time. Life-course factors also influence compensatory processes that are engaged to meet cognitive challenge, and to ameliorate the adverse effects of structural and functional decline. The revised model is discussed in relation to recent lifespan and longitudinal data as well as emerging evidence about the effects of training interventions. STAC-r goes beyond the previous model by combining a life-span approach with a life-course approach to understand and predict cognitive status and rate of cognitive change over time.

## Introduction

Decades of behavioral research in the latter part of the 20th century characterized a variety of age-related cognitive deficits including memory problems, executive processing dysfunction and declines in speed of processing that typify normal older adults (e.g., Craik and Salthouse 2000). Despite volumes of performance data and numerous theoretical advances (e.g., Schaie et al. 1996; Schaie and Willis 2011a, b; Birren and Schaie 2005), a coherent integrated account of cognitive aging based on behavioral data alone proved to be elusive. Fortunately, the end of the last century also brought major developments in in vivo human neuroscience methods, most critically, functional and structural imaging that permitted scientists to relate neural activity and structural brain measurements to specific cognitive processing abilities (Cabeza et al. 2005). Additional and more recent advances in imaging of white matter pathways, amyloid deposits, connectivity patterns, genetic, pharmacological and other biomarkers have provided a wealth of new indices of neurophysiological status that can be integrated with behavioral performance assessments to identify the neurocognitive underpinnings of typical age-related decline (Grady 2008; Buckner et al. 2009; Bäckman et al. 2006; Raz and Lustig 2014; Laukka et al. 2013).

In 2009 we published a model, which we referred to as the Scaffolding Theory of Aging and Cognition—“STAC” for short (Park and Reuter-Lorenz 2009). STAC aimed to explain age differences in cognitive function by incorporating the effects of a broad range of adverse biological and neurophysiological factors that had been associated with normal aging to date, and to delineate their dynamic interaction with protective factors and newly emerging, putative compensatory processes deemed to be at work in the older brain. While the model was originally developed in the context of cross-sectional studies comparing extreme groups of younger and older adults, it incorporated principles that were likely to be at play across the lifespan. The goal of the present review is to re-evaluate and revise STAC in view of new meta-analyses, lifespan (i.e., cross-sectional across adulthood) and longitudinal data that have been published since the model was conceived. We also consider new evidence about the effects of cognitive training and lifestyle factors in the context of STAC—these were identified as “future issues” in 2009, as relevant data were limited at that time.

## Overview: The Scaffolding Theory of Aging and Cognition (2009)

**Fig. 1:**
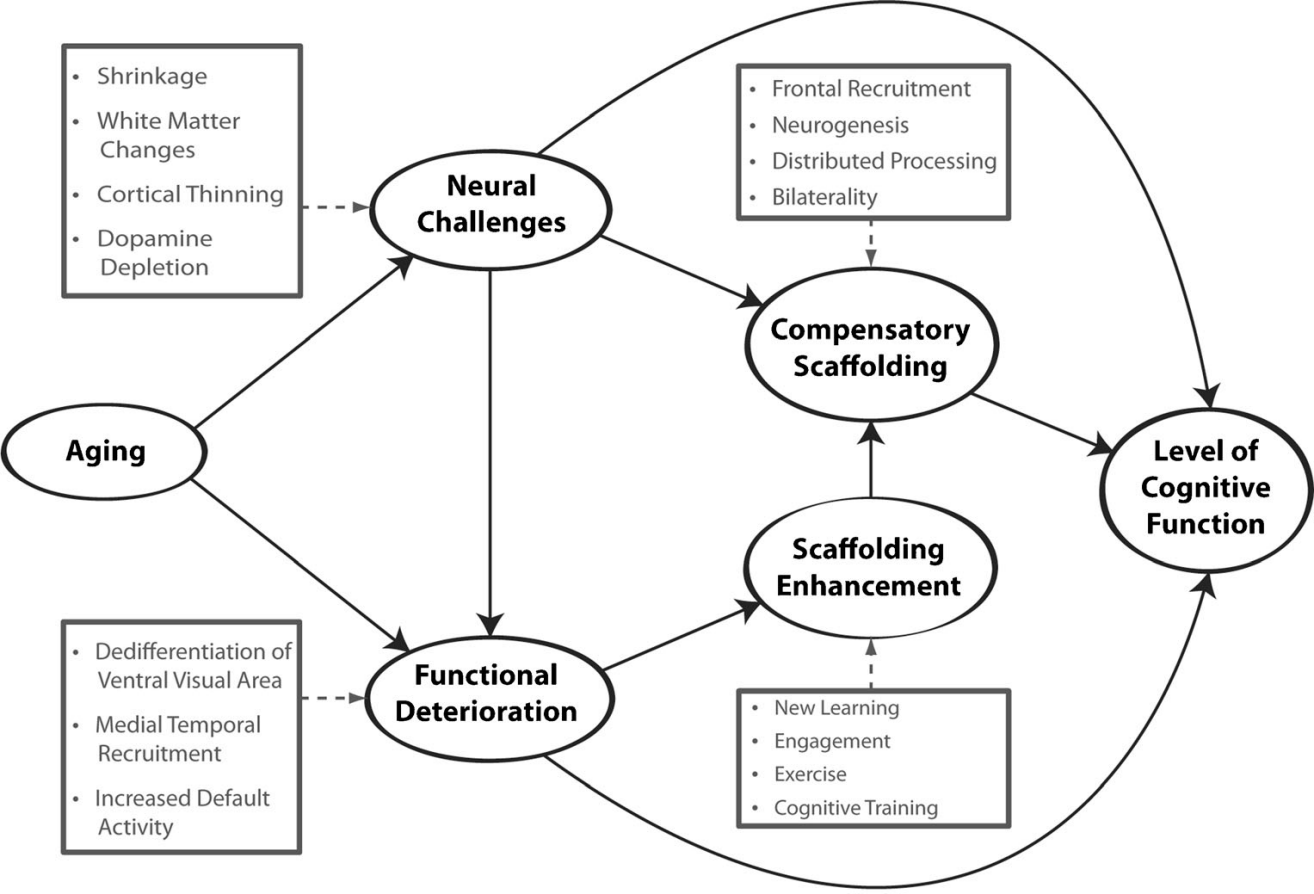
A conceptual model of the scaffolding theory of aging and cognition (STAC) (Park and Reuter-Lorenz 2009)

The STAC model as originally conceived and depicted in Fig. 1, includes the following basic principles to explain an older individual’s level of cognitive function. First, relative to younger adults, healthy older adults are affected by varying degrees of neural degradation, which were categorized as “neural challenges” and “functional deterioration,” respectively. Neural challenges are primarily structural changes in the brain that occur with age, including cortical thinning and regional atrophy, loss of white matter integrity, and dopamine depletion. Functional deterioration refers to indicators of maladaptive, age-related brain activity that have been very well documented in the imaging literature including dedifferentiation (decreased specificity) of ventral-visual and motor areas (Park et al. 2004; Voss et al. 2008; Bernard and Seidler 2012), decreased memory-related recruitment of medial temporal lobe regions (Cabeza et al. 2004; Gutchess et al. 2005) and dysregulation of the default mode network (Lustig et al. 2003; Persson et al. 2007; for a review, see Park and Reuter-Lorenz 2009; Reuter-Lorenz and Park 2010).

Second, according to STAC, the level of cognitive function an individual displays is a consequence of these negative indices, combined with a beneficial process, which we term “compensatory scaffolding.” Compensatory scaffolding operates to ameliorate or counteract the adverse effects of neural and functional decline, and can be considered a form of “positive” plasticity that accompanies aging, whereas the adverse changes in brain structure that occur with age are negative forms of plasticity (Cramer et al. 2011; see also, Greenwood 2007). More specifically, scaffolds entail the engagement of supplementary neural circuitry that provides the additional computational support required by an aging brain to preserve cognitive function in the face of localized or global neurofunctional decline. Indications of compensatory scaffolding evident in the neuroimaging literature include greater activation or additional recruitment of prefrontal brain regions (Gutchess et al. 2005; Davis et al. 2008), compared to young adults, an effect now documented in parietal regions as well (e.g., Angel et al. 2011; Huang et al. 2012). Overactivation can also take the form of bilateral recruitment, where older adults activate left and right brain (sometimes homologous) regions on tasks for which younger adults show lateralized activity (Cabeza 2002; Reuter-Lorenz et al. 1999; Reuter-Lorenz et al. 2000; Tyler et al. 2010; Cappell et al. 2010; Schneider-Garces et al. 2010; for a review see, Cabeza and Dennis 2012). Meta-analytic evidence has now verified the pervasiveness and reliability of age-related overactivation in cross- sectional studies of younger and older adults, across a wide range of task domains including perceptual, memory and executive function tasks (Spreng et al. 2010). We note speculatively that neurogenesis, while more limited in older adults, is also a potential source of positive plasticity that may contribute to compensatory scaffolding (Fuchs and Flügge 2014; Lovden et al. 2013).

STAC represents the brain as a dynamically adaptive structure that changes in both positive and negative ways with age. Figure 1 indicates that both neural challenge and deterioration will stimulate scaffolding, which in turn moderates the effects of deleterious brain influences on cognitive performance. While typical age-related changes in brain structure and function can stimulate compensatory scaffolding, very severe deterioration can eventually undermine the brain’s ability to provide effective compensation. Finally, the model suggests that it is possible to enhance neural scaffolding activity by some explicit interventions that include various lifestyle activities including exercise, intellectual engagement and new learning, as well as more formal cognitive training interventions.

STAC was proposed as a neurofunctional account detailing the effects of age on cognition, most of which had been established from cross-sectional studies that compared extreme age groups of younger and older adults. However, continuous intra-individual lifespan principles related to compensatory scaffolding are inherent in the theory. In particular, the notion of scaffolding itself comes from cognitive development and skill acquisition research, which has demonstrated that existing mental abilities can be harnessed as support for the acquisition of new ones. The work of Petersen and colleagues (Petersen et al. 1998) was especially influential in the development of STAC. Most important was their observation that during early stages of skill acquisition, a network including prefrontal regions was very active, but activity in these regions decreased as performance became more skilled and activity increased in new skill-specific regions of the brain. They interpreted the initial but transient set of activations as providing scaffolding for the acquisition of novel skills, with the activation shifting elsewhere as skill increased. The ideas for STAC also drew upon evidence that with greater task demand, also considered a form of neural challenge, younger adults show increased activation of primary task regions, recruitment of additional brain regions or both, typically involving regions of prefrontal cortex that mediate executive functions (Reuter-Lorenz and Lustig 2005; Reuter-Lorenz and Cappell 2008). These lines of evidence suggested that the brain possesses particular adaptive neurocognitive “strategies” that are manifested under conditions of cognitive and behavioral challenge, and that similar mechanisms can be adopted with age to preserve established skills or to maintain optimal performance.

The STAC model as depicted in Fig. 1 accounts for individual differences in level of cognitive functioning at one specific time point, presumably in later adulthood. Over the past 5 years there has been an increase in new data sets concerning neurocognitive function in middle age, longitudinal age-related change, and intervention studies indicating later-life plasticity in response to experience. The goal of the present paper is to revisit STAC, and the scaffolding construct, and to re-evaluate the model in light of new developments in the field. We are particularly concerned with addressing emerging evidence about longitudinal influences on neural structure and function across the lifespan. We also consider new evidence about the effects of genetics, health, experiential and life-style variables on cognition, as these approaches were not integrated into the original STAC model. Based on these considerations, we propose a revised model, “STAC-r,” that integrates new data and knowledge about neurocognitive aging.

## Compensatory Scaffolding

The concept of compensatory scaffolding is at the heart of the original STAC model. The notion that compensatory and supportive neural mechanisms enable maintenance of cognitive function with age has received considerable support since the model was developed (e.g., Berlingeri et al. 2010; Burzynska et al. 2013; Davis et al. 2012; Geerligs et al. 2012; Vallesi et al. 2011; Nyberg et al. 2014; Chanraud et al. 2013; Davis et al. 2008), although the indicators and mechanisms of compensation continue to be debated (Cabeza and Dennis 2012; Fabiani 2012). Because compensatory scaffolding is a key component retained in the revised model, we first review some of its properties in light of recent emerging evidence and other concepts that have come to the fore since 2009, and then introduce the revised model.

### Brain Maintenance

According to STAC, neural challenge in the form of neurophysiological deterioration or neural insults that come with age, rather than age itself, are the impetus for compensatory scaffolding. Therefore and quite logically, older individuals who maintain a youthful neurobiological status, through favorable genetics, environmental factors, lifelong pro-health behaviors and beneficial lifestyle activities (Hillman et al. 2008; Josefsson et al. 2012; Vemuri et al. 2012) will need less compensatory scaffolding and reorganization. This axiom is captured by the term “brain maintenance” (Nyberg et al. 2012), an elegant and important construct that argues that a key characteristic of successful aging is simply the absence of age-related pathology. In terms of brain activation, one would expect that older adults who exhibited preservation of cognitive function in some domains would show more “youth-like” brain patterns, and minimal overactivation, and other age-specific neural indicators potentially characteristic of less compensatory scaffolding.

Several recent studies relating performance to fMRI activity and other measures of neurophysiological integrity bear this out (e.g., Nagel et al. 2011; Rosano et al. 2012). For example, using brain activation patterns obtained during a picture memory encoding task, Duzel and colleagues (2011) identified a subgroup of older adults whose activity profiles were virtually indistinguishable from those associated with encoding success in younger adults (Duzel et al. 2011). This “youth-like” older subgroup had recollection memory performance that was also indistinguishable from the younger group, while showing no evidence of prefrontal over-activity often taken to be compensatory. Thus, their preserved memory was more likely due to preserved neurobiology than to compensation. Consistent with this interpretation, the older subgroup whose encoding-related activation deviated most from the youth-like pattern had poorer memory, along with more default network dysregulation, and region-specific gray matter loss. Interestingly, Duzel et al. (2011), as the STAC model would predict, also identified a subgroup of older adults with preserved memory and overactivation of prefrontal and parietal regions relative to the younger group, which may have provided compensatory support for weaker engagement of other memory-dedicated circuitry. Their data, in accordance with STAC, suggest that older adults may achieve preserved cognition by means of preserved neurobiology, compensatory processes, or some combination of these factors.

If a hallmark of successful cognitive aging is maintenance of abilities and underlying neurobiology, then its assessment requires longitudinal measurements to evaluate the degree of change over time. Data of this type is just beginning to emerge. One important study (Pudas et al. 2013) classified individuals (ages 55–75) as having preserved memory versus average memory decline over a prior 15–20 year period, and used fMRI to assess their current brain activity profiles during a memory task. The comparison group with average memory decline had lower hippocampal activity than the successful agers, and a young comparison group. The successful group had greater hippocampal activity and more prefrontal activity (including left and right inferior frontal gyrus, IFG) than both young and average-old comparison groups (c.f., Persson et al. 2012). The two groups of older adults showed no differences in regional brain volumes or white matter integrity. So, as the authors point out, while overactivation in the successful agers is consistent with compensation, greater prefrontal and hippocampal engagement might have been characteristic of these people since an early age. More studies of this type are needed to clarify the contributions of brain maintenance and compensatory processes to sustained levels of high performance over time.

### Brain Efficiency

Another notion entailed in the compensatory scaffolding component of STAC is *efficiency* of brain function (Duverne et al. 2008; Rypma et al. 2006; Rypma et al. 2007; Rypma and D’Esposito 1999; see also Neubauer and Fink 2009). Brain efficiency figures into the conceptualization of STAC in two important ways that have been advanced by research over the past 5 years (cf. Poldrack 2014). First, neurophysiological decline can lead to reduced efficiency, meaning that the rate and/or quality of neural processing (e.g., signal to noise ratio, fidelity of representations, speed of neural transmission) is reduced in association with aspects of perceptual and memory encoding, dedifferentiation, and poor default network regulation. As noted above, accumulating evidence continues to support these sources of dysfunction (Carp et al. 2011; Bernard and Seidler 2012; Garrett et al. 2013; Barulli and Stern 2013). In addition, structural decline, in the form of gray matter loss for example, could also be associated with reduced efficiency, resulting in a compensatory response in associated networks.

Recent work by Tyler and her colleagues (Tyler et al. 2010; Meunier et al. 2014; Shafto et al. 2012) is especially relevant for linking age-related neural decline to compensation and adaptivity of language processing networks. They report, for example, that older adults show marked gray matter loss in left lateralized regions specialized for syntactic processing in young adults, especially left IFG (Tyler et al. 2010). The greater the age-related volume loss in this region, the greater the recruitment of right IFG (and right temporal regions) and the more correlated its activity with left IFG. Critically, performance on syntactically demanding tasks was found to be age-equivalent, consistent with the idea that the recruitment of right hemisphere circuitry provides compensation for the declining left hemisphere regions specialized for language.

Gray matter reductions are not always detected in older adults however, due perhaps to methodological factors or subject variability, and atrophy does not always or fully correspond with regions that show activation changes measured with fMRI (e.g., Kalpouzos et al. 2012; Maillet and Rajah 2013; Chen et al. 2011). Furthermore, as Poldrack (2014) has pointed out, univariate fMRI indices of reduced or increased activation are ambiguous with respect to the “energy expenditure” of the neural system. While investigating the effect of parametric variations in task demand on activation levels can help to interpret group differences in activation levels (Reuter-Lorenz and Cappell 2008), multimodal imaging approaches and network analyses will become increasingly useful for clarifying how aging affects the efficiency of neural systems, and in turn drives compensation.

The second way that the idea of efficiency is relevant to STAC pertains to the efficiency of scaffolded networks. According to the model, while compensatory processes are proposed to assist (or attempt to assist) with computations mediated by the primary network, they are less efficient than primary networks in their youthful state. New evidence consistent with this proposition (Meunier et al. 2014) again comes from studies of language. Using fine-grained analyses of gray matter density Meunier et al. (2014) observed localized decreases that were interpreted to drive changes in network functional connectivity including the recruitment of additional right hemisphere circuitry during syntactic processing (Meunier et al. 2014). Moreover, using graph theoretic connectivity measures of network efficiency, Meunier and his colleagues showed that with these additional regions, network efficiency was lower in older adults. In this case, greater involvement of right hemisphere regions was also associated with poorer syntactic processing, raising questions about whether compensation is the most fitting interpretation of the function being served by these areas (Meunier et al. 2014) or whether dedifferentiation is more accurate.

### What Neural Processes are Meditated by Compensation?

A fundamental question that arises in relation to the notion of scaffolding and compensation more generally is: What processes are being carried out by the additional regions or circuitry recruited to support the primary network? Are these additional regions assisting with the same neural computations conducted by the dedicated areas, and therefore the same cognitive strategies, do they provide alternative routes to achieve the same strategy, or do they mediate different strategies altogether, as has been suggested in some language domains (Shafto et al. 2012)? These interesting and fundamental questions are highly unlikely to have the same answer for every task circumstance or for each cognitive domain in which scaffolding may be evident, but the STAC-r model is sufficiently flexible to allow for such differences.

There are indications, for example, that some cognitive processes may be aided by recruiting domain-general executive control and working memory circuitry, providing a way to “off-load” high neural demands when a task requires high resource expenditure (e.g., Simmonds et al. 2008). A meta-analysis of response inhibition tasks in young adults confirmed a dominant role of right IFG in this process, but in addition found that for tasks with complex rules, right dorsolateral prefrontal cortex (PFC) regions were also active (Simmonds et al. 2008). Critically, when rule complexity has been varied, older adults have been found to recruit additional PFC regions during response inhibition tasks at lower levels of demand than younger adults (Vallesi et al. 2011). Related effects have been observed in the context of semantic processing, where additional domain-general executive control may be recruited by high-performing older adults (Peelle et al. 2013). These effects resemble the recruitment of additional prefrontal circuitry by older adults at lower levels of working memory task demand (compensation-related utilization of neural circuits hypothesis, CRUNCH, Reuter-Lorenz and Cappell 2008; see also Cappell et al 2010; Schneider-Garces et al. 2010). Multi-voxel pattern analyses have demonstrated that additional recruitment by older adults may indeed reflect greater reliance on domain-general resources at lower levels of demand than younger adults (Carp et al. 2010). In many instances however, there is insufficient information within a particular study to infer what functions are being served by additional regions of activity (or heightened connectivity) in older adults, so answers to these questions await future research.

Research in the future is also likely to focus further on individual differences in the use of scaffolding for particular cognitive functions, and to clarify further the conditions under which scaffolding is advantageous, especially given the likelihood that that the best cognition and healthiest brains will be associated with minimal structural degradation and little need for compensatory activity (de Chastelaine et al. 2011). It will also be important to determine whether there is selective vulnerability to aging of different parcellated brain systems (Wig et al. 2014) or large-scale brain networks (Bressler and Menon 2010), as patterns of compensatory scaffolding are likely to be systematically related to the magnitude and locus of neural degradation. Along this line, recent work from the Park lab investigating a large lifespan sample of adults from age 20 to 89 (Park et al. 2013) found evidence that task-activated fronto-parietal regions associated with successful subsequent memory, showed age differences earlier in the lifespan than task negative regions associated with the default network. Thus, different networks may have different trajectories of age-related decline (see also, Grady et al. 2010). Park et al. (2013) also found that low ability adults showed differences in task negative activity early in the lifespan whereas high ability adults maintained levels of neural activation until old age (see also, Daffner et al. 2011). These findings generally support the importance of individual difference variables, such as cognitive ability, in understanding the range of neural activity associated with compensatory activations, as well as the importance of lifespan studies to fully understand the developmental trajectory of neurocognitive aging (see also, Nyberg et al. 2010).

## STAC-r: A Revised Model of the Scaffolding Theory of Aging and Cognition

The longitudinal trajectories of neural and cognitive change and variables that promote brain maintenance and decline are beginning to figure more prominently in studies of neurocognitive aging. Moreover, there is increasing interest in neural and cognitive function in middle age, which likely sets the stage for the course of aging later in life (Karlamangla et al. 2014). For example, it is important to understand to what extent cognitive status in late adulthood is determined by neurofunctional status and reliance on compensatory processes in early and middle adulthood (e.g., Borghesani et al. 2012; Macpherson et al. 2014; Schaie and Willis 2011a, b; Willis et al. 2010; Thambisetty et al. 2013). Do middle-aged adults who rely on compensation earlier in life than their age-matched peers go on to age more poorly, given that they show older-age brain function at a young age?

**Fig. 2:**
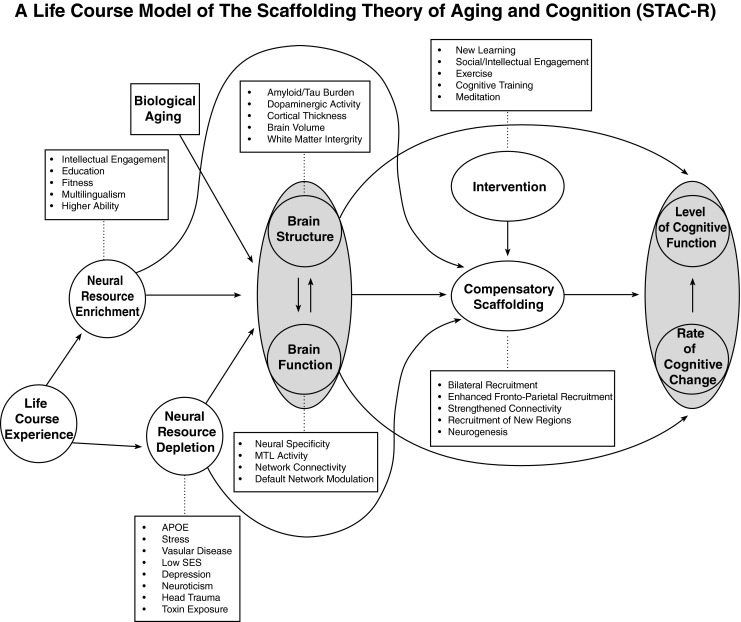
A conceptual model of the scaffolding theory of aging and cognition-revised (STAC-r)

The STAC model predicts cognitive function at a single time point during an individual’s lifespan with a focus on later-life cognition. This was partially because, with “aging” itself as the primary input to the model, it was not possible to afford a role for experience, genetics, and environment to influence the course of aging and, in turn, level of cognitive function. The increasing evidence that these broad factors are important determinants of the trajectories of neural and cognitive function (e.g., Agrigoroaei and Lachman 2011; Albert et al. 1995; Bender and Raz 2012; Anstey and Cherbuin 2012; Anstey 2008; Boron et al. 2012; de Frias et al. 2014; Stiehler et al. 2009; Zanjani et al. 2013) provides a sound basis for revising STAC to recognize the life-course influences on neurocognitive aging. Thus the revised model, which we refer to as “STAC-r”, now incorporates life-course variables that impact structure and function of the aging brain (see Fig. 2). We use the term "life course” to mean the accumulation of experiences and states an individual has experienced from birth to death (Mayer 2002). The model indicates that both life-span (aging) and life-course (experience) variables impact the structure and function of the brain and also directly affect the development of compensatory scaffolding, a construct that retains the core features from the original model that were described above. The next sections, consider the new components of the STAC-r model.

### Predicting Cognitive Function with STAC-r

Before discussing predictors of cognitive function in STAC-r, it is essential to define the outcomes it predicts. The original model, shown in Fig. 1, depicts “Level of Cognitive Function” as the predicted outcome that refers to an individual’s overall cognitive status as a consequence of all of the predictor variables. In STAC-r, shown in Fig. 2, the reach of the model is increased to encompass the life course by incorporating the outcome variables of "Rate of Change in Cognitive Function” along with “Level of Cognitive Function.” Rate of change provides a metric of the steepness of cognitive decline over time, which of course influences the level of cognitive function. We also considered that the level of cognitive function could conceivably influence the rate of change, but omitted this from the model because that relationship is currently uncertain (e.g., Gow et al. 2012; Salthouse 2013, 2012). Perhaps one simplification in the model is the treatment of cognitive function as an undifferentiated global construct, when in fact different domains of cognition may age differentially within and across individuals (Mungas et al. 2010; Van Petten et al. 2004). It is also important to recognize that, although at the group level longitudinal data will often be characterized by linear decline, there is considerable inter-individual variability and almost certainly, nonlinearity in individual trajectories (Boyle et al. 2013; Dixon and de Frias 2009). That is, intra-individual cognitive aging is frequently characterized by both plateaus and declines across the life course of a given individual (Dixon and de Frias 2009). STAC-r allows for nonlinearity of decline due to differential amounts and timing of neural degradation both within and between individuals. For example, such nonlinearity might occur when an individual who showed little change in cognition for a prolonged period from say, ages 52 to 64, might show a sharp, nonlinear decline in function after a heart attack, chemotherapy, or diagnosis of some other serious illness.

### Introducing Two New STAC-r Constructs: Neural Resource Enrichment and Neural Resource Depletion

A major limitation of the original STAC model is that it did not allow for positive and negative influences extraneous to the brain to shape age-related neural reorganization and cognitive response. A more elaborate model is required to address new evidence about how life-course influences can contribute to neural health or neural dysfunction (Harris and Deary 2011; Habib et al. 2007; Josefsson et al. 2012; Kauppi et al. 2014; Cherbuin et al. 2009; Eramudugolla et al. 2014). STAC-r includes two new constructs that represent the combined contributions of life experiences, genetic endowments, and environmental influences that operate to either enhance or deplete brain resources.

One construct—“*neural resource enrichment*”—includes influences that act to enhance brain structure or function. A considerable amount of correlational data suggests that individuals who are engaged in intellectual and social activities in middle and late adulthood fare better cognitively than less engaged peers. For example, self-reports of higher participation in cognitive, leisure, and social activities are related to better cognitive ability in middle-aged and older adults, less age-related cognitive decline and are even associated with a decreased risk of being diagnosed with Alzheimer’s Disease (Amieva et al. 2010; Hall et al. 2009; Plassman et al. 2010; Reed et al. 2011; Singh-Manoux et al. 2003; Stern 2009; Wilson et al. 2013). Likewise, there is growing evidence that a high level of education and/or ability may be protective of cognitive function, and that individuals who are highly educated tend to be diagnosed with dementia at later ages than less educated or lower ability adults (Amieva et al. 2014; Bennett et al. 2003; Christensen et al. 2009; Karlamangla et al. 2009; Yaffe et al. 2009). In addition to these variables, high levels of cardiovascular and physical fitness (Head et al. 2012; Colcombe et al. 2003, 2004; Erickson et al. 2013b, 2010), bilingualism (Bialystok et al. 2007, 2012; Gold et al. 2013; Schweizer et al. 2012), multilingualism (Alladi et al. 2013) and engaging in enriched leisure activities (Wilson et al. 2002b, 2002a; Landau et al. 2012) have all been identified as having beneficial outcomes for cognitive aging.

The model suggests two paths by which such beneficial and protective effects can operate. First, neural enrichment could directly enhance or preserve brain structure and function by promoting efficient connectivity, increasing cortical thickness, synaptic density and other indicators of brain health. As an example, there is a great deal of convergent evidence that a high level of cardiovascular fitness will modestly enhance brain health directly, most likely through increased secretion of brain-derived neurotrophic factor (BDNF; Erickson et al. 2013a), or improved vasculature (Thijssen et al. 2010; Chapman et al. 2013). In this case, the fit individuals are more likely to have healthier and better functioning brains than unfit adults, and greater protection against risk (Head et al. 2012).

The second pathway is less direct, in that life course enrichment factors could increase the capacity for compensatory scaffolding providing additional protection against the expression of cognitive decline in the face of neural insults that occur with older age. For example, in some individuals high levels of education (or other enrichment factors) may not prevent age-related decline of brain structure and function, but rather enable enhanced scaffolding, so that despite neural degradation, cognitive function continues to be high. It follows from this example that high ability or highly educated older adults with normal cognitive function could harbor greater neural degradation than their less educated counterparts (Stern 2012, 2002). In fact, that is what researchers have reported (Brickman et al. 2008, 2012, 2009; Rentz et al. 2009). This type of finding provides strong evidence for compensatory processes like neural scaffolding and cognitive reserve (Barulli and Stern 2013) that can provide additional neural and cognitive support in the face of structural decline and neural challenge.

Aspects of STAC-r have parallels to the concepts of brain reserve and cognitive reserve advanced by Stern (Stern 2012; Barulli and Stern 2013). Brain reserve refers to properties of brain structure that confer tolerance to pathology and would presumably be indicated by highly favorable values on the structural and functional brain indices portrayed in Fig. 2 (Stern 2012). Cognitive reserve refers to cognitive processes or compensatory cognitive strategies engaged to cope with pathology, which are related to overall level of cognitive ability (Stern 2012). Both forms of reserve can be enhanced by the enriching variables outlined in STAC-r, and likewise the depleting variables (see below) would presumably diminish both forms of reserve. Because compensatory scaffolding in STAC and STAC-r refers to neural processes, some functional indicators of scaffolding may also be neural correlates of cognitive reserve; although it’s not clear that cognitive reserve has the developmental origins and on-going (life-long) utility in the face of cognitive challenge that we attribute to scaffolding. Moreover, scaffolding is considered a dynamic response of the neural system to decline, and the ability to compensate for neural degradation through the kind of plastic reorganization that is central to the STAC models is less clearly represented in the reserve models. Thus, STAC-r provides a dynamic model where both neurophysiological variables and compensatory neural processes operate jointly to predict cognitive function over time.

In sum, neural resource enrichment is an ongoing process that confers beneficial neural resources across brain structure, function and compensatory potential, depending upon the nature of the enrichment variable and the overall condition of a given brain. It is worth noting that, most likely, there is a broad range of additional potential influences that could enhance brain structure, function and/or scaffolding, including familial, social and emotional relations, among other variables (e.g., Antonucci et al. 2014; Hershfield et al. 2013). The cognitive enrichment variables in STAC-r are not intended to be exhaustive, but rather represent major candidates for neural resource enrichment.

A second construct—“*neural resource depletion*,” constitutes negative influences on brain structure, neural function and ultimately cognition, as shown in Fig. 2. As with enriching influences, the factors listed here are intended to be representative. One factor that has an especially powerful depletion effect on cognition is the presence of the APOE-4 gene. Even one copy of the gene, which is carried by roughly 20 % of the population, substantially increases the risk of an Alzheimer’s diagnosis in one’s lifetime (by a factor of 1.7; (Slooter et al. 1998)). There is clear evidence, as well, that amyloid and tau deposition, which are comprised of the plaques and tangles associated with Alzheimer’s disease (AD), have subtle but readily measurable negative effects on cognition in healthy, cognitively “normal” adults (Rodrigue et al. 2012, 2009; Mielke et al. 2014). Vascular risk factors, such as smoking, obesity and diabetes, also have adverse impacts on brain health increasing signs of cerebrovascular injury, regional atrophy, and cognitive dysfunction (Debette et al. 2011; de Frias et al. 2014; Bender and Raz 2012). Moreover, there is an association of heart disease with amyloid deposition (Honjo et al. 2012) and major depression is associated with both a heightened white matter and amyloid burden (Brickman et al. 2009). Stress may play a key role in hippocampal shrinkage as a result of hyper-secretion of cortisol, which provides short term mobilization of neural resources, but is destructive of hippocampal tissue over the long term (McCune 2007). Thus, a diversity of factors subtly affect brain integrity, but it is may not be until late adulthood that the aggregation of these insults exert a measurable effect on cognition.

When the STAC model was first published recent advances in vivo amyloid imaging techniques had just been developed. These techniques are now used in many laboratories that study mild cognitive impairment (MCI) and AD, and even a few labs (e.g., Park Aging Mind Lab) where the focus is on mechanisms of cognitive health and well-being. The initial amyloid imaging data are congruent with autopsy data and show that at least 20 % of seemingly healthy adults aged 60 and over carry elevated levels of brain amyloid (Hedden et al. 2012; Mormino et al. 2011, 2008; Rodrigue et al. 2012; Aizenstein et al. 2008; Pike et al. 2007), with the possibility that they may already be in a state of “preclinical AD,” which can be detected many years before symptoms emerge (Sperling et al. 2011). The trajectory of decline is not yet clear for seemingly healthy aging, cognitively normal individuals with a high amyloid burden. Longitudinal data are quite limited due to the very recent development of in vivo amyloid imaging. Therefore, a critically important question is whether all asymptomatic individuals high in amyloid burden will go on to develop AD, and, if so, with what the latency? Further, might life-course factors of neural resource enrichment or depletion influence that latency, as the STAC-r model would predict? In line with this, recent evidence suggests that APOE-4 carriers appear to be protected from the effects of high amyloid burden if they report high engagement in cognitively-stimulating activities in middle age (Jagust and Mormino 2011).

It is clear that the multifactorial nature of STAC-r and that the many variables that can enrich and deplete neural resources are vast, ranging from such influences as poor air quality, as a potential influence on depletion (Weuve et al. 2012; Bhatia 2012), to mindfulness practice as a potential influence on enrichment (Zeidan et al. 2010). In sum, there are many routes to good and poor brain health, some predetermined by genetics whereas others such as exercise, nutrition, and intellectual engagement are, to varying extents, under control of the individual. Just as risk factors for heart disease have been identified and guide individuals toward healthy heart behaviors, neuroscientists are beginning to make similar progress on brain aging, greatly assisted by remarkable new imaging tools, techniques, and ligands that allow for measurement of amyloid, tau, and dopamine receptors.

### Brain Structure and Brain Function

In the original STAC model, brain structure and function were represented by neural challenge and functional deterioration, both of which were affected only by the adverse influences of aging. By taking a life-course perspective, STAC-r (Fig. 2) depicts the possibility that brain structure and function may change bi-directionally, reflecting both positive and negative effects of plasticity, development and life-course influences. Moreover, maintenance of brain structure and function over time is possible depending on the age of the individual, and the balance of enriching and depleting life-course influences. STAC-r now incorporates these principles. The indices of structure and function listed in Fig. 2, can reflect neurofunctional status, or with multiple measurements, the magnitude of neurophysiological change over time.

Multimodal imaging is a key focus of much present aging research, as it permits a more complex understanding of how different dimensions of the brain physiology operate jointly to affect cognitive function. Continued use of multimodal imaging will be necessary to understand how the risks associated with different combinations of biomarkers, including metabolic measures, volumetric indices, ligand binding etc., combine to affect brain network structure, function, and cognition (e.g.(Hedden et al. 2012; Walhovd et al. 2010)). While STAC-r makes no explicit claims about the relative potency of different types of brain degradation in orchestrating the transition from healthy to pathological aging, consideration of the relative contributions of different biomarkers may be a productive approach in further understanding cognitive function.

In particular, studies that integrate the effect of the most predictive structural biomarkers with functional biomarkers would be particularly productive in understanding mechanics underlying cognitive decline. An influential model of progression towards AD (Jack, et al. 2010; Jack et al. 2013) suggests that the earliest signs of preclinical AD is amyloid deposition, which may have a lengthy latency before frank behavioral symptoms of AD appear. The next variable most likely to be observed is an increase in tau proteins in the cerebrospinal fluid, followed by declines in hippocampal volume. This cascade of events ultimately leads to AD. Developing a sophisticated understanding of neural events that unfold temporally in healthy adults will provide deeper understanding of the brain processes that control age-related changes in cognition, just as detailed characterization of change over time has resulted in a richer understanding of the transition to AD.

### Interventions

Both the original STAC model and STAC-r incorporate the potential benefits of formal, structured interventions, which according to both models, enhance compensatory scaffolding and ultimately cognitive function. STAC-r also incorporates the possibility that interventions could potentially have a direct influence on brain structure and function. For example, anodal transcranial direct current stimulation has recently been shown to alter frontal activation and improve cognitive task performance in older adults (Meinzer et al. 2013). To the extent that cognitive interventions are effective, cognition itself can influence neural plasticity and scaffolding, and this possibility is also represented in the STAC-r model.

Most training research in the scientific community has been focused on enhancing specific cognitive abilities in older adults and the impact of training on both the trained ability (specific transfer) and overall cognitive function (general transfer). With respect to the STAC-r model, the most relevant studies are those that have included neuroimaging data. Neural data, particularly fMRI approaches, are needed to understand how the aging brain has changed after a substantive intervention, and what form the changes take. While the mechanisms of training are largely unknown, training could cause fundamental changes in neural structure through volumetric increases and network connectivity, or it could affect compensatory mechanisms.

The most ambitious training intervention conducted to date on older adults was a large, multi-site trial enrolling over 2800 participants (Ball et al. 2002) with participants receiving extended training to improve speed of processing, memory function, or reasoning. Results indicated that the training improved older adults’ performance on the domain in which they were trained and that the effect persisted 5 years later (Willis et al. 2006), but that there was no domain-general improvement in cognitive function. There was also evidence for self-reported improvement in tasks of everyday living (Willis et al. 2006) and speed of processing training improved depressive symptoms over 5 years (Wolinsky et al. 2009). Most recently, a 10-year follow-up of the participants provided evidence that those who were trained showed better performance than controls in their ability to perform Instrumental Activities of Daily Living (Rebok et al. 2014). These studies suggest that relatively limited and focused cognitive experiences can have a long-term effect on cognition and activities of daily living, but additional research is needed to understand the mechanisms underlying these effects.

An increasing number of studies are examining the impact of cognitive training on neural structure and function in older adults (Lustig et al. 2009; Lustig and Reuter-Lorenz 2012; Lovden et al. 2010b; Kirchhoff et al. 2012; Belleville et al. 2011; Backman and Nyberg 2013; Brehmer et al. 2011; Heinzel et al. 2014), and whereas some interventions are effective, benefits are not always found. For example, Dahlin et al. (2008) reported an increase in striatal function after training the updating function in working memory for young, but not older adults, who also showed minimal performance benefits. In contrast, Anguera et al. (2013) reported a promising EEG study demonstrating that older adults who played a multi-tasking video game showed enhancements on multi-tasking that exceeded the performance of untrained young adults, and the effects endured for 6 months. Moreover, facilitation effects for untrained cognitive control tasks were observed. The behavioral improvements were associated with enhanced midline frontal theta power and frontal-posterior theta coherence, providing insight into the mechanism underlying these positive results (Anguera et al. 2013). The findings support the likelihood that frontal regions are an important locus for compensatory processes and provide evidence supportive of STAC-r. Other recent computerized intervention studies find that training-related improvements in older adults are associated with reduced activity in regions that were overactive pre-training, or more youth-like activation patterns post-training, suggesting that training can also improve the efficiency of neural function (Heinzel et al. 2014; Meinzer et al. 2013).

Another approach taken to interventions is to immerse or engage participants in an environment designed to promote scaffolding through cognitive challenge, and determine whether sustained exposure to the enriched environment creates new scaffolding and maintains or even improves cognition (e.g., Stine-Morrow et al. 2008). In a recent study (Park et al. 2014), participants learned to quilt or learned digital photography for 15 h a week for 14 weeks. These cognitively demanding conditions were compared to control conditions where subjects engaged in group activities for the same amount of time, but no new skills were learned and the focus was on discussions, nonintellectual field trips, and other group activities. Another control group worked at home on low-demand paper and pencil tasks, and a final group was a non-treatment control. The results indicated that the two groups who engaged in cognitive challenge (learning quilting, photography, or both) performed better on measures of episodic memory than the low challenge groups.

What mechanisms underlie these observed improvements in cognition? As Nyberg et al. (2012) recently stated, “Even though the empirical evidence of an association between an enriched lifestyle and cognitive performance in aging is convincing, we hasten to add that the brain mechanisms mediating this association are unknown.” (p. 301). We hypothesize that engagement in complex learning tasks or an enriched life style forces a great deal of self-initiated processing that can stimulate plasticity and create neural scaffolds (see also, Lovden et al. 2010a). Early work in the Park lab demonstrated that guiding older adults to perform mental operations and use strategies that they would not spontaneously initiate improved episodic memory (Cherry et al. 1993; Cherry et al. 1996; Smith et al. 1998). Novel activities in which participants become deeply engaged require a marked increase in self-initiated processing and sustained activation of executive function, memory, and reasoning which in turn creates new neural paths (scaffolds) that ultimately facilitate cognition. This speculative hypothesis could be readily tested with neuroimaging obtained at multiple time points to identify the mechanisms underlying the benefits from such late-life experiences. We also note that one advantage of engagement research is that the “dosage” of the intervention can be hundreds of hours, whereas cognitive training typically involves a fraction of this level of exposure. It is truly urgent to invest in understanding how brain activity and structure are affected by active, engaged lifestyles.

## Conclusions and Future Directions

Like STAC, STAC-r is a conceptual model that combines adverse and favorable influences on brain structure and function to determine cognitive status. Whereas STAC focused on adverse effects of aging together with beneficial effects of compensation, STAC-r incorporates life-course influences that enhance, preserve, or compromise brain status, compensatory potential, and ultimately cognitive function over time. The model is compatible with concepts of reserve and posits that like compensatory scaffolding these can be affected in positive or negative ways by factors that enrich or deplete neural resources. Given the vast complexity of the human brain and the huge quantities of neurobiological now being accrued, the interpretative challenge confronting neuroscientists is immense. We hope that the STAC and STAC-r models can provide fertile roadmaps for hypothesis development and testing. Here we suggest some questions and directions for future research.

1. An important challenge for the field is to understand how strongly different extrinsic factors modify trajectories of aging, the developmental time course of such influences and, relatedly how much plasticity exists in the aging brain at different stages of the life course. For example, some enriching influences may be greatest in youth, whereas others may be most effective at middle age. Might there be others that yield their greatest advantages in older age?
2. Brain imaging continues to give rise to new measures of brain structure and function, such as resting state connectivity, multivoxel pattern analysis, and measures of network variability. It is not yet known which measures are most informative about the mechanisms underlying cognitive function, nor do we know which measures are most sensitive predictors of cognitive change over time. While STAC-r treats these brain indices as roughly equivalent, future research can help to clarify their relative sensitivity to different life- course influences and their relation to compensatory processes that can maintain optimal cognitive function. One suggestion is that increased focus on resting state networks may be especially informative as they are more likely to reflect actual changes in brain mechanisms, rather than task-based strategy differences due to age or induced by an intervention. The application of new measures to assess network efficiency will also advance our understanding of the functional properties of declining and reorganized networks.
3. Along these lines, future research may be able to identify brain patterns characteristic of neural health and neural decline. STAC-r predicts that the ability to maintain youthful levels of brain structure and function over time, relying minimally on compensatory scaffolding, will be associated with greatest efficiency and minimal cognitive decline. Longitudinal brain and performance data obtained over a period a decade or more, would likely permit neuroscientists to test these predictions and ultimately predict future cognitive status effectively based on brain measurements obtained at an earlier age. It is noteworthy that major pathologic indicators of brain health account for considerably less than 50 % of variation in the rate and onset of cognitive decline (Boyle et al. 2013). New constructs incorporated in STAC-r can provide some direction towards identifying and quantifying variables that influence neural and cognitive outcomes.
4. Apart from life course influences on the trajectories of aging, it is critical to understand whether and how interventions applied later in life can forestall or reverse cognitive decline. STAC-r posits a two-fold influence: on compensatory scaffolding and on brain status; however little is currently known about the mechanistic bases for training effects, or the factors that limit training success, especially in older adults. Training could improve the efficiency of the primary network, leading to less reliance on compensatory processes (Lustig et al. 2009), it could improve compensatory scaffolding, and the extent to which these mechanisms operate may depend on age, and the nature of the intervention. Future studies that combine training interventions with neural measurements across the life span will help to address these issues.
